# Assessment of osteoporosis knowledge and its determinants among tuberculosis patients in tertiary care hospital Malaysia: A prospective study

**DOI:** 10.1016/j.jctube.2024.100416

**Published:** 2024-01-24

**Authors:** Zohra Bhatti, Madeeha Laghari, Amer Hayat Khan, Bandeh Ali Talpur, Syed Azhar Syed Sulaiman

**Affiliations:** a Department of Pharmacy Practice, Kulliyyah of Pharmacy, International Islamic University, Malaysia; b School of Public Health, Physiotherapy and Sports Science, University College Dublin, Ireland; c School of Pharmaceutical Sciences, Universiti Sains Malaysia, Malaysia; d School of Computer Science ans Statistics, Trinity College Dublin, Ireland

**Keywords:** Tuberculosis, Osteoporosis, Osteoporosis knowledge test, Malaysia

## Abstract

**Objective:**

Osteoporosis self-care knowledge is essential to encourage the patient’s contribution towards controlling the disease. Osteoporosis incidence with various infectious diseases prompts us to conduct a study on osteoporosis knowledge among Tuberculosis (TB) patients. This study aimed to assess osteoporosis-related knowledge and its influencing factors among TB patients.

**Methods:**

A prospective cross-sectional study was conducted in the TB clinic of Hospital Pulau Pinang, Malaysia, and an osteoporosis knowledge test (OKT), a structured questionnaire, was used to collect data. TB patients aged 18 years and above with compromised bone health were included in the present study. Overall knowledge scores were dichotomized, calculating the mean score, descriptive statistics, and multivariate regression analysis was used to determine the influence of risk factors on knowledge scores.

**Results:**

Of 337 patients, 129(38.28 %) had good osteoporosis knowledge and 208(61.72 %) had poor knowledge. Among them, 231(68.54 %) were males, and 106(31.45 %) were females, with a mean age of 46.5 ± 17.1 years. The mean ± SD of OKT total score was 10.5 ± 2.0. The mean ± SD of the exercise knowledge score was 5.3 ± 3.4, while the nutrition knowledge score was 5.0 ± 3.2. Male gender (OR 3.86, 95 % CI 1.98–7.53), low-income level (OR 1.92, 95 % CI 1.12–3.30), rural residents (OR 2.49, 95 % CI 1.46–4.27) and participants with no formal education (OR 4.34, 95 % CI 1.11–16.84) or ≤12 years of education (OR 3.63, 95 % CI 1.52–8.65) were significantly responsible for poor OKT score.

**Conclusion:**

The results indicated that most TB patients had a poor perception of osteoporosis. More extensive strategies should be developed to enhance the campaign of awareness programs among TB patients to improve preventive measures of osteoporosis, such as calcium intake and exercise.

## 1 Introduction

Osteoporosis, a disease characterized by reducing bone mass and weakening of bone microstructure, compromised bone strength, and intensifying the risk of fracture, is a growing health hazard around the world. The prevalence of osteoporosis was 10.6 % among the Malaysian male population [1]. Another study revealed that 15.3 % of Malaysian Chinese aged ≥40 years and 32.6 % of those aged 71 years had osteoporosis [2]. Approximately 90 per 100,000 hip fracture incidence rate was reported annually in Malaysia [3].

A study conducted in a university hospital in Zürich has reported association of respiratory TB with bone fracture, even without comorbidities or steroid use, bone degrading was reported in 3––5 % of active TB cases [4]. In a retrospective study conducted in Taiwan, the risk of osteoporosis was presented as 1.36-folds higher among respiratory TB as compared to non-respiratory TB patients. The overall incidence of osteoporosis was 9.61 per 1000 person-years among respiratory TB, as reported by the National Health Insurance Research Database of Taiwan [5]^.^ The osteoporosis incidence rate was 2.2 % among the TB patients and 1.1 % in non-TB patients. Similarly, female TB patients were more likely at risk of osteoporosis than male TB patients [6]. Another retrospective study conducted in Korea reported a 58.6 % prevalence of osteopenia and 14.6 % of osteoporosis among TB patients [7].

The prevalence rate of above-mentioned comorbid conditions reveals TB patients at high risk of osteoporosis. This comorbid condition urges us to attempt a survey to know about the knowledge level of this disease among TB patients. Various osteoporosis studies primarily focus on female gender, pre- and postmenopausal conditions, and adults in different communities. Osteoporosis knowledge assessment studies have been conducted with diverse comorbid conditions like cancer [8], thalassemia [9], HIV [10], and diabetes [11], [12]. The data on osteoporosis knowledge assessment among TB patients is scarce. However, to our knowledge, the current study is the first to evaluate the level of osteoporosis awareness among TB population. Knowledge assessment of osteoporosis disease is vital for controlling and improving patients' health outcomes. Osteoporosis knowledge assessment was found to be correlated with age, education, and income [13]. An Iranian study indicated that higher education is significantly associated with better knowledge [14]. Similarly, an average knowledge about associated demographic risk factors, smoking, extra coffee intake, physical activity, education and living conditions was reported in Lublin, Poland [15].

Knowledge of potential health complications is a prerequisite for engaging in health-promoting practices. More particularly related to low bone density is the knowledge of dietary, exercise and lifestyle characteristics that contribute to development of osteoporosis. The investigation about osteoporosis among TB patients can offer a unique and ground-breaking facts that enhance our understanding on the association of TB disease and osteoporosis. This study has ability to shed light on the interplay between two diseases and linked implications for patient care and public health. Moreover, the present study contributes to a more holistic approach to patient care by identifying the comprehensive care and better patient outcomes. The insights obtain from this study can provide the base for developing preventive strategies and guidance on lifestyle changes like nutrition and exercise.

The current study aimed to access the knowledge of osteoporosis among TB patients by using OKT, an internationally validated scale, to design better policies for improving osteoporosis awareness and preventive health measurements among TB patients in Malaysia. However, to achieve this goal, it is necessary to first examine and comprehend the basic awareness of TB patients towards osteoporosis. The findings can direct the development of future interventions and educational material to encourage strategies that can potentially slow down and control the osteoporotic complications and improve TB patients' quality of life.

## 2 Methods

### 2.1 Study settings and population

This was a descriptive cross-sectional study conducted in the respiratory clinic of Hospital Pulau Pinang; a public sector tertiary care hospital that covers a large proportion of the Pinang population in Northern Malaysia. In this study, the population was comprised of patients diagnosed with drug-susceptible PTB aged ≥18 years who were taking their treatment for more than 30 days. After bone screening, they were also diagnosed with the compromised bone health condition (osteopenia means T-score lies between 1 to −2.4 and osteoporosis means T-score is >−2.5). Around 90 % patients were having osteopenia and 10 % were osteoporotic. These were not taking any medication to treat this condition. Those patients who could communicate, understand the questions, and provide oral informed consent, were eligible for inclusion. However, patients with drug-resistant TB and physical and/or cognitive impairment were excluded from the study because of the complexities of managing drug-resistant TB cases and the need for specialized care.

### 2.2 Sample size and sampling technique

The required sample size was calculated using the Cochrane formula. Based on the assumption of a 95 % confidence interval, a 5 % margin of error, and a 10 % non-respondent rate, the total calculated sample was 430. Of 430 randomly selected patients, 337 (78.5) accepted to participate in the study. Of those who refused to participate, 39(41.93 %) reported that they had no time to participate, 31(33.33 %) had a lack of interest, whilst 23(24.73 %) did not explain. The questionnaires were distributed in the waiting room for about 20–30 min.

### 2.3 Development of data collection tool

In the present study, the data was collected using tOKT revised version 2012, which was used with the permission of the developer [16]. The socio-demographic factors including gender, age, education, smoking habit, residence, income level, marital status, employment status, hospital visitors, were collected by self-designed questionnaire. Numerous osteoporosis knowledge studies have utilized the OKT 2012 version (used in the current study) and have shown acceptable reliability and validity. Notably, Malay translated was used and validated in type II diabetic patients in the same settings in Malaysia [11]. Thus, this tool was culturally appropriate and understood by the study participants. The data tool was basically in English and translated into Malaysian by linguistic experts, with forwarding and backward translation. It was pre-tested among 10 physicians and 10 nurses in the respiratory clinic, 10 medical academia experts and updated as required. All domains of scales displayed good internal consistency. The reliability coefficients of the OKT tool (Malay version) were 0.8, which was well within the acceptable limits (Cronbach’s α > 0.7). During the pre-test, the questionnaire was evaluated for its clarity, accuracy, reliability, sensitivity of the subject matter, and cultural acceptability in the region. Before distributing the questionnaire, the oral consent for participating in this study was taken from each patient.

### 2.4 Scoring

The OKT comprises 32 multiple choice instruments comprising two Knowledge domains: nutrition and exercise. It was designed to measure knowledge of osteoporosis and prevention strategies related to exercise and calcium intake. The two subscales had 14 items in common. The total score for the OKT scale ranges from 0 to 32. The range for the nutrition subscale was from 0 to 26, while that for exercise went from 0 to 20. Responses were recorded as correct or incorrect to calculate the good and poor knowledge scores. If respondents indicated more than one option for a question or if a question remained unanswered, the question was defined as incorrect. In scoring, the question was given one mark for the correct answer, while each inaccurate or unknown answer was marked as 0. The overall knowledge score was obtained by summing up these responses. The patients' knowledge was classified into high and low groups using a cut-off point of 14, based on the previous validation study of OKT- Malay version [11]. Consequently, those with a total score equal to or below the mean were classified as having poor knowledge, whereas those above the mean were considered to have good knowledge.

### 2.5 Statistical analysis

For statistical data analysis SPSS 24 was used in the present study. Before data entry, manual data cleaning checks were used to identify unreadable marks on questionnaires, blank questions, and wrong coding. After completing the manual check process, frequencies were presented for each response and evaluated for missing answers. Descriptive statistics using frequency distribution tables were used to summarize socio-demographic characteristics and to compute total knowledge scores. Multiple regression analysis was done to study the association between poor knowledge level and demographic factors.

## 3 Results

### 3.1 Demographic and clinical characteristics of the participants

The socio-demography of patients is given in Table 1. Of 337 patients, 231(68.54 %) were males, and 106(31.45 %) were females, with a mean age of 46.5 ± 17.1 years. The majority of participants (56.67 %) were living in rural areas. Most of them (69.14 %) reported being currently married. For the level of education, 268(79.52 %) had ≤12 years of education. The proportion of employment was high (72.70 %), but majority of participants (61.72 %) had low monthly income (<2000 RM).

**Table 1:** Demographical characteristics of study participants.

Characteristics	Patients n = 337(%)
**Gender**	
Male	231(68.54)
Female	106(31.45)
**Mean age ± S.D (years)**	46.5 ± 17.10
**Age range(years)**	
18–35	92(27.29)
36–50	103(30.56)
51–65	90(26.71)
>65	52(15.43)
**Race**	
Malays	137(40.65)
Chinese	152(45.10)
Indians	37(10.97)
Others	11(3.26)
**Education**	
No formal education	25(7.41)
≤12 years	268(79.52)
>12 years	44(13.05)
**Smoking**	
Yes	188(55.78)
No	149(44.21)
**IVDU**	
Yes	39(11.57)
No	298(88.43)
**Residence**	
Rural	191(56.67)
Urban	146(43.32)
**Income Level**	
<2000	208(61.72)
3000–5000	129(38.28)
**Marital Status**	
Single	72(21.36)
Married	233(69.14)
Divorce	32(9.49)
**Employment Status**	
No	92(27.29)
Yes	245(72.70)
Patient Category	
General population	312(92.58)
Prisoners	25(7.42)

### 3.2 Analysis of OKT scores

Most of the study participants had poor scores in the OKT subscale about osteoporosis knowledge (Table 2). 24(7.12 %) of patients correctly answered, *“Eating a diet low in dairy products causes high chances of osteoporosis”.* For the question, “*Parents/grandparent’s osteoporosis makes them more prone to get osteoporosis*”, 114(33.83 %) of participants checked the “more likely”, 66(19.58 %) chose “less likely”, 85(25.22 %) indicated “Neutral response”. 116(34.42 %) correctly responded to the statement, *“Being a White or Asian woman increases the chance of getting osteoporosis”*. 77(22.84 %) respondents correctly answered for, *“Smoking daily can cause osteoporosis”*. About 215(63.79 %) participants chose “*Walking briskly”*; 248 (73.59 %) considered *“jogging or running as a helpful exercise for reduction of osteoporosis”.* Only 125(37.09 %) respondents knew “*Broccoli as the best source of calcium*”, and 81(24.03 %) accurately chose *“Canned sardines as the best source of calcium”.* Most participants had poor knowledge regarding the nutrition subscale (Table 2).

**Table 2:** Detail description of OKT questions with frequencies and percentage of correct and incorrect responses.

Variables	Percentages n (%)
**Risk factors of osteoporosis**:	
Do the following make it more or less likely that someone will develop osteoporosis	
**Eating a diet LOW in DAIRY products can cause osteoporosis**	
***More likely***	24(7.12)
Less likely	191(56.67)
Neutral	95(28.19)
Don’t know	27(8.01)
**Being menopausal change of life**	
***More likely***	106(31.45)
Less likely	107(31.75)
Neutral	91(27.00)
Don’t know	33(9.79)
**Having a parent or grandparent who has osteoporosis**	
***More likely***	114(33.83)
Less likely	66(19.58)
Neutral	85(25.22)
Don’t know	72(21.36)
**Being a white or Asian woman**	
***More likely***	116(34.42)
Less likely	64(18.99)
Neutral	115(34.12)
Don’t know	42(12.46)
**Being elderly Man**	
***More likely***	199(59.05)
Less likely	55(16.32)
Neutral	41(12.16)
Don’t know	42(12.46)
**Having ovaries surgically removed**	
***More likely***	79(23.44)
Less likely	83(24.62)
Neutral	114(33.8)
Don’t know	61(18.10)
**Taking cortisone (steroids) for a long time**	
***More likely***	104(30.86)
Less likely	70(20.77)
Neutral	114(33.82)
Don’t know	49(14.54)
**Being overweight**	
More likely	140(41.5)
***Less likely***	90(26.70)
Neutral	51(15.13)
Don’t know	56(16.61)
**Having an eating disorder**	
***More likely***	103(30.56)
Less likely	78(23.14)
Neutral	84(24.92)
Don’t know	72(21.36)
**Consuming more than two alcoholic drinks per day**	
***More likely***	91(27.00)
Less likely	122(36.20)
Neutral	91(27.00)
Don’t know	33(9.79)
**Smoking daily**	
***More likely***	77(22.84)
Less likely	103(30.56)
Neutral	85(25.22)
Don’t know	72(21.36)
**To strengthen bones, it is recommended that a person exercise at a moderately intense level for 30 min a day at least**	
3 days a week	33(9.79)
4 days a week	55(16.32)
***5 days a week***	200(59.34)
Don’t know	49(14.54)
**Exercise makes bones strong, but it must be hard enough to make breathing**	
Just a little faster	83(24.62)
***Much fast, but talking is possible***	50(14.84)
So fast talking is not possible	100(29.67)
Don’t know	104(30.86)
**Which Activity is the best way to reduce osteoporosis**	
Swimming	82(24.33)
***Walking briskly***	215(63.79)
Stretching	14(4.154)
Don’t know	26(7.72)
**Which Activity is the best way to reduce osteoporosis**	
Bicycling	122(36.20)
Yoga	87(25.82)
***Lifting weights***	51(15.13)
Don’t know	77(22.84)
**Which Activity is the best way to reduce osteoporosis**	
***Jogging or running for exercise***	248(73.59)
Golfing using golf craft	14(4.15)
Gardening	30(8.90)
Don’t know	45(13.35)
**Which Activity is the best way to reduce osteoporosis**	
Bowling	42(12.46)
Doing laundry	17(5.04)
***Aerobic dancing***	165(48.96)
Don’t know	113(33.53)
**Which one of the following is the best source of calcium**	
Apple	45(13.35)
***Cheese***	201(59.64)
Cucumber	13(3.85)
Don’t know	78(23.15)
**Which one of the following is the best source of calcium**	
Peanut butter	54(16.02)
Turkey	76(22.55)
***Canned sardines***	81(24.03)
Don’t know	126(37.38)
**Which one of the following is the best source of calcium**	
Chicken	68(20.17)
***Broccoli***	125(37.09)
Grapes	35(10.38)
Don’t know	109(32.34)
**Which one of the following is the best source of calcium**	
***Yogurt***	205(60.83)
Strawberries	20(5.93)
Cabbage	29(8.60)
Don’t know	83(24.63)
**Which one of the following is the best source of calcium**	
***Ice cream***	58(17.21)
Grapefruit	112(33.23)
Radishes	56(16.62)
Don’t know	111(32.93)
**What is the recommended calcium intake for an adult**	
600 mg to 800 mg daily	45(13.35)
***1000 to 1200 mg daily***	71(21.07)
1400 mg to 1600 mg	36(10.68)
Don’t know	185(54.89)
**How many glasses of milk an adult should take per day**	
1 glass daily	12(3.56)
2 glass daily	152(45.10)
***3 or more glasses daily***	93(27.59)
Don’t know	80(23.74)
**What is the best reason for taking a calcium supplement**	
If a person skips breakfast	23(6.83)
***If a person does not get enough calcium***	164(48.66)
If a person is over 45 years old	61(18.10)
Don’t know	89(26.41)
**Which vitamin is required for the absorption of calcium**	
Vitamin A	35(10.38)
Vitamin C	199(59.05)
***Vitamin D***	55(16.32)
Don’t know	48(14.24)
**Which is the best source of the vitamin required for the absorption of calcium**	
Carrot	46(13.65)
Oranges	38(11.28)
***Sunlight***	113(33.53)
Don’t know	140(41.54)
**Which is the best source of the vitamin required for the absorption of calcium**	
Spinach	71(21.06)
Cheese	137(40.65)
***Salmon***	52(15.43)
Don’t know	77(22.85)
**Which of the following is the recommended amount of the vitamin required for the absorption of calcium for an adult, 50 years old and older**	
**800**–**1000 IU daily**	63(18.69)
1200–1400 IU daily	56(16.62)
1600–1800 IU daily	55(16.32)
Don’t know	163(48.36)
**When is the best time to build strong bones**	
Childhood	51(15.13)
***Adolescence***	73(21.66)
Young adulthood	71(21.06)
Don’t know	142(42.14)
**Osteoporosis can be diagnosed by**	
Blood test	102(30.27)
***DXA scan***	52(15.43)
Symptoms	85(25.22)
Don’t know	98(29.08)
**Once you have osteoporosis,**	
There is nothing you can do	76(22.55)
***You can take medication to treat it***	180(53.41)
You must be careful lifting objects	49(14.54)
Don’t know	32(9.49)

Correct answers were given in bold and italic.

Of the study participants, 129(38.23 %) patients had good, and 208(61.72 %) had poor OKT knowledge. The mean ± SD of OKT total score was 10.5 ± 2.0. The mean ± SD of the exercise knowledge score was 5.3 ± 3.4, while that of the nutrition knowledge score was 5.0 ± 3.2.

**Fig. 1:**
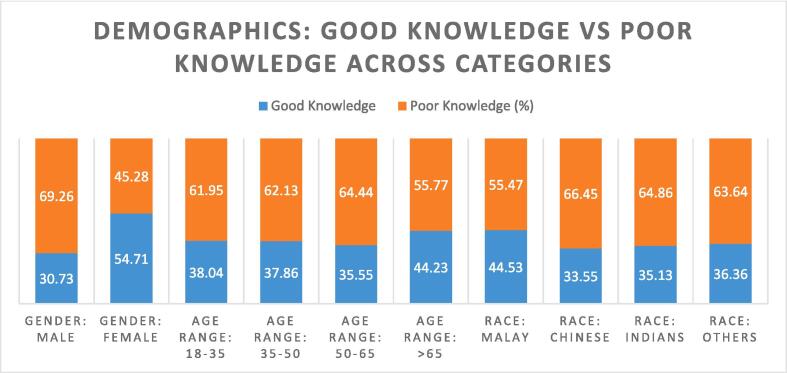
Scores of osteoporosis knowledge scale examined by demographic characteristics.

**Fig. 2:**
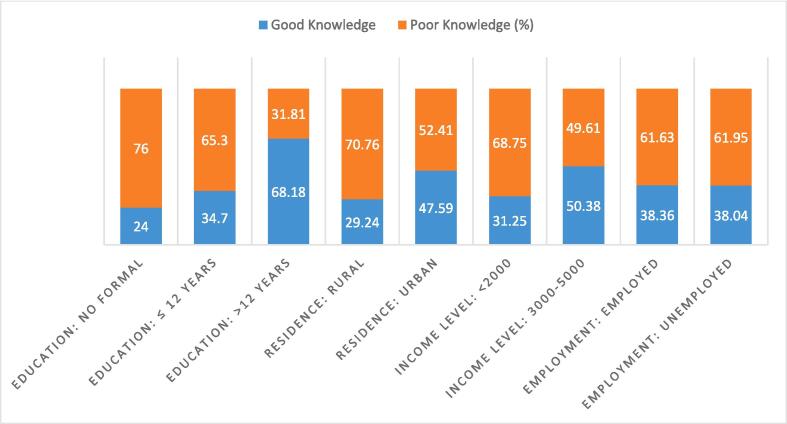
Socio-economic factors: good knowledge vs poor knowledge across category.

**Fig. 3:**
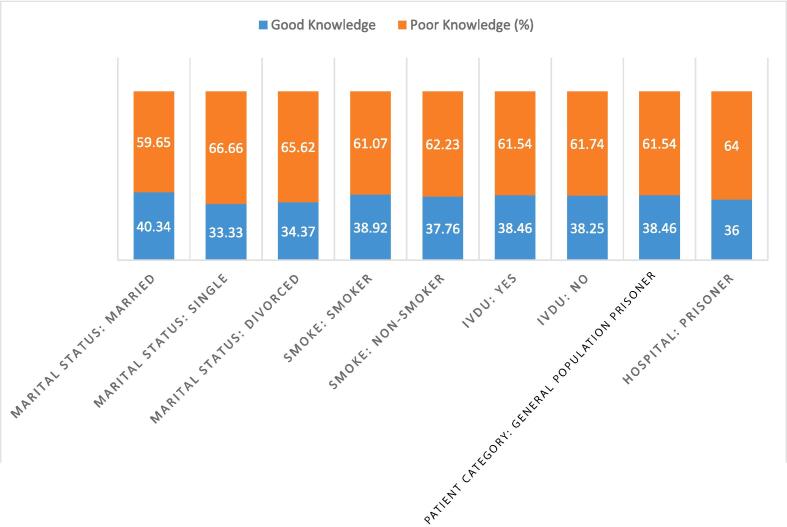
Marital status, Habits and other: good knowledge Vs poor knowledge across categories.

Fig. 1, Fig. 2, Fig. 3 depicted the percentage of knowledge score of study participants against socio-dedemographic characteristics.. Around 54.71 % females and 30.73 % males had good knowledge about osteoporosis. The 66.0 % TB patients who lies in the age range more than >65 years had high score of knowledge. Malaysian population had good knowledge than other races (Fig. 1).

Study participants with highest education (68.2 %) and those having high income (50.38 %) had better awareness compared to other groups (Fig. 2).

In Table 3, multiple regression analysis was performed to assess the influencing factors of poor OKT scores. Male gender (OR 3.86, 95 % CI 1.98–7.53), low-income level (OR 1.92, 95 % CI 1.12–3.30), rural residents (OR 2.49, 95 % CI 1.46–4.27) and participants with no formal education (OR 4.34, 95 % CI 1.11–16.84) or ≤12 years of education (OR 3.63, 95 % CI 1.52–8.65) were significantly responsible for poor OKT score.

**Table 3:** Multiple regression analysis of responsible factors of osteoporosis knowledge scores with demographic characteristics.

Characteristics	*β*	S.E	OR (95 % CI)	*P-value*
**Gender**				
Male	1.35	0.34	3.86(1.98–7.53)	<0.001
Female	−1.35	0.34	0.25(0.13–0.50)	
**Age range(years)**				
18–35	0.82	0.52	2.27(0.81–6.35)	0.11
35–50	0.48	0.44	1.63(0.67–3.91)	0.27
50–65	0.20	0.44	1.22(0.51–2.95)	0.64
>65	−0.59	0.42	0.55(0.24–1.26)	0.16
**Race**				
Malays	−1.29	0.91	0.27(0.04–1.64)	0.15
Chinese	−1.14	0.93	0.31(0.05–1.98)	0.22
Indians	−1.91	0.98	0.14(0.02–1.01)	0.05
Others	1.31	0.91	3.70(0.62–22.09)	0.15
**Education**				
no formal	1.46	0.69	4.34(1.11–16.84)	0.03
≤12 years	1.28	0.44	3.63(1.52–8.65)	0.04
>12 years	−1.46	0.68	0.23(0.06–0.88)	0.03
**Income level**				
Low	0.65	0.26	1.92(1.12–3.30)	0.01
High	−0.65	0.26	0.52(0.30–0.89)	
**Residence**				
Rural	0.91	0.27	2.49(1.46–4.27)	0.01
Urban	−0.91	0.27	0.40(0.23–6.83)	
				
**Smoke**				
Yes	−0.52	0.29	0.59(0.32–1.05)	0.07
No	0.52	0.29	1.69(0.94–3.03)	
**IVDU**				
Yes	−0.10	0.43	1.10(0.46–2.62)	0.81
No	−0.10	0.43	0.90(0.38–2.13)	
Patient Category				
General population	−0.14	0.49	0.86(0.32–2.29)	0.77
Prisoners	0.14	0.49	1.15(0.43–3.07)	

## 4 Discussion

This study was conducted to assess osteoporosis knowledge among TB patients in Malaysia. According to significant study findings, 61.72 % of TB patients had a lack of osteoporosis knowledge. In line with the current study the knowledge about the source of calcium was reported 50 % [17]. Similarly, several studies consistent with current research reported low exercise knowledge [18], [19]. In another study, the majority of participants incorrectly answered the questions on the OKT scale. The mean OKT score indicated poor osteoporosis-related knowledge [12].

The current study differs from previously conducted studies in certain aspects. First, the present study was conducted among TB patients to evaluate their osteoporosis-related knowledge. Secondly, a scoring system was developed for OKT, and participants' scores for each domain were analyzed and correlated with various socio-demographic factors.

Demographic characteristics have shown that most of respondents were Chinese, and the majority were 35–50 years old. Among racial distribution, Chinese population ratio 152(45.10 %) was higher followed by Malays 137(40.65 %) and Indians 37(10.97 %). Besides that, 79.52 % have an average educational background, which is consistent with another study conducted in Malaysia [20]. The osteoporosis information, knowledge, understanding, and perception about exercise and calcium sources were poor (62.0 %). In line with our results, other studies show more than 50.0 % of patients have correct knowledge about exercise for bone health [19] and, therefore, require preventive educational training programs to increase awareness about osteoporosis [21], [22], [23]. Poor knowledge among current study participants depicts a low perception of seriousness towards osteoporosis. Although knowledge is a crucial component in promoting self-care, knowledge alone still cannot result in the practice of preventive behavior or a positive attitude [24]. So, it is necessary to perceive the seriousness of osteoporosis among TB patients. Literature has shown that people believe osteoporosis is an inevitable factor of ageing and do not take it as a severe disease that may even cause death [25], [26]. In the case of osteoporosis knowledge among TB patients, the results of our study indicate that 54.7 % of total females had good knowledge on osteoporosis than males (Fig. 1), which is consistent with an Indian study where women were reported to had higher knowledge than men [27]. While males had significantly poor knowledge score (OR 3.86, 95 % CI 1.98–7.53), which would be explained by postulating that osteoporosis in men is a relatively unknown topic and has been described rarely. The osteoporotic fracture risk in men is roughly one-third of women, but the fatality risk following the fracture is 1.6 times higher in men [15].

TB patients with poor knowledge of osteoporosis indicated that majority of participants were not well educated. Study participants with no formal education (OR 4.34, 95 % CI 1.11–16.84) or ≤12 years of education (OR 3.63, 95 % CI 1.52–8.65) were significantly responsible for poor OKT scores (Table 3). Patients with >12 years of education have a good proportion of knowledge, supporting our finding that educational level was reported as a significant factor in a few studies [28], [29]. Furthermore, rural residents had been associated with low knowledge of osteoporosis [30]. In line with the present study, rural residency (OR 2.49, 95 % CI 1.46–4.27) significantly influences the unawareness of osteoporosis knowledge among TB patients [21]. This could be because , the training campaigns for disease awareness are mainly carried out in the megacity or overpopulated areas in developing countries. This practice might become the decisive reason for the low level of knowledge in rural areas. In our results, a significant association was found between low-income level (OR 1.92, 95 % CI 1.12–3.30) and poor knowledge of osteoporosis (Table 3), as supported by the literature [11], [31].

The findings of this study strongly indicate the need to develop policies and conduct community-based programs mainly focused on increasing public awareness and knowledge of the disease. Critical interventions are compulsory to improve attitudes towards osteoporosis among TB patients. On observing the rising trend of osteoporosis in different age groups, it is necessary to enhance the role of general physicians to raise awareness regarding osteoporosis at various health clinics and centres. Current study findings can be used as scientific evidence to help design and provide adequate health education.

A limitation inherent in most knowledge assessment studies is the cross-sectional study design and sampling techniques that could have created some bias. We conducted this study with limited resources. Future studies must include a more extensive sample of TB patients from different health centres and tertiary hospitals in Pinang to explore more accurate and in-depth results. In addition, future studies are needed to shed light on daily practices of patient exertion and nutritional approaches, such as protein and calcium intake, to avoid more health complications.

## 5 Conclusion

Overall, TB patients had poor osteoporosis knowledge. Implementing awareness and educational programs among TB patients might increase preventive practices and measures toward osteoporosis. Such preventive procedures need to focus on calcium-rich nutrition and regular exercise.

## Availability of data and materials

The datasets used and analyzed during the current study available from the corresponding author on reasonable request.

## Consent for publication

Not applicable.

## Funding

No funding.

## Ethical approval

The study was approved by the Medical Research Ethics Committee (MREC), Ministry of Health, and Malaysia (Registration ID: NMRR-18-1145-40397; MREC reference: dim. KKM/NIHSEC P18-1198(6).

## Authors contribution

Zohra Bhatti: Conceptualization, Methodology, Investigation, Writing-original draft, Data Curation, Formal analysis, Investigation Madeeha Laghari Conceptualization, Methodology, Writing- review and editing, Visualization. Amer Hayat Khan supervised this project. Conceptualization, Project administration, Investigation, Supervision, Validation. Syed Azhar Syed Sulaiman Project administration, Resources, Supervision. Bandeh Ali Talpur Formal analysis, Funding acquisition. Software, Validation.

## Declaration of competing interest

The authors declare that they have no known competing financial interests or personal relationships that could have appeared to influence the work reported in this paper.
